# Early Detection of Hyperdense Basilar Artery Signs Through Comparison With Previous Images

**DOI:** 10.7759/cureus.66135

**Published:** 2024-08-04

**Authors:** Tatsuya Tanaka, Takahiro Kumono, Hiroshi Itokawa, Akira Matsuno

**Affiliations:** 1 Department of Neurosurgery, International University of Health and Welfare, Narita Hospital, Narita, JPN

**Keywords:** hyperdense basilar artery sign, hyperdense artery sign, basilar artery occlusion, computed tomography, acute ischemic stroke

## Abstract

The presence of the hyperdense basilar artery (HDBA) sign, which indicates basilar artery occlusion (BAO), plays an important role in the early diagnosis and intervention in patients with acute ischemic stroke. However, qualitative and quantitative assessment of the HDBA sign is challenging. This case report describes a 60-year-old woman with a history of diabetes mellitus, hypertension, and cerebral infarction. She developed progressive loss of consciousness and ataxic respiration. A noncontrast-enhanced head computed tomography (CT) scan performed three hours after symptom onset revealed the HDBA sign compared with previously obtained CT images. Quantitative measurements revealed a significant increase in Hounsfield units (HUs) in the basilar artery. Subsequent three-dimensional CT angiography confirmed the occlusion of the vertebrobasilar artery. This case highlights the importance of comparing current and previous imaging findings in detecting the HDBA sign. Quantitative HU measurements may further aid diagnosis. Early detection of the HDBA sign on noncontrast-enhanced head CT is critical for expediting the diagnosis and treatment of BAO.

## Introduction

The hyperdense artery sign on noncontrast-enhanced computed tomography (CT) is critical for diagnosing large vessel occlusions. Although the hyperdense basilar artery (HDBA) sign is also useful for diagnosing basilar artery occlusion (BAO), significant interexaminer variability exists [1]. The HDBA sign is less established than the hyperdense middle cerebral artery (MCA) sign because of concerns about posterior fossa artifacts that alter vessel density and the absence of a corresponding artery for symmetry assessment. In this case report, the comparison with a previous head CT proved valuable in identifying the HDBA sign and making a diagnosis of BAO

## Case presentation

**Figure 1:**
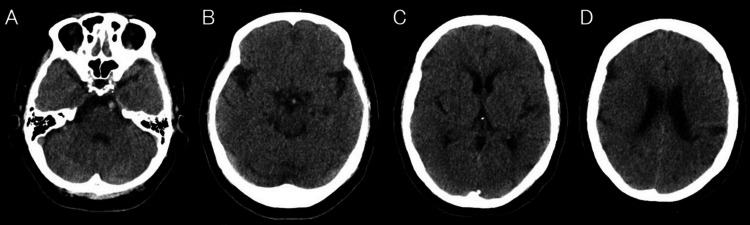
(A-D) Initial noncontrast-enhanced head CT scan performed three hours after symptom onset reveals no hemorrhage or early ischemic changes. (A,B) The basilar artery appears hyperdense CT: computed tomography

A 60-year-old woman with a history of diabetes mellitus, hypertension, and a lacunar infarction of the left corona radiata two months prior presented with a sudden onset of dizziness and vomiting. The patient was taking aspirin to prevent cerebral infarction. Upon arrival, approximately 90 minutes after symptom onset, her Glasgow Coma Scale score was 13; however, it deteriorated to 6, with significant oral secretions and a decline in respiratory status, resulting in percutaneous oxygen saturation of 60%. Two hours after symptom onset, a neurological examination revealed a progressive loss of consciousness and ataxic respiration. Emergency tracheal intubation and mechanical ventilation were initiated. A noncontrast-enhanced head CT scan performed three hours after symptom onset revealed no hemorrhage or early ischemic changes; however, the basilar artery appeared hyperdense (Figure 1).

**Figure 2:**
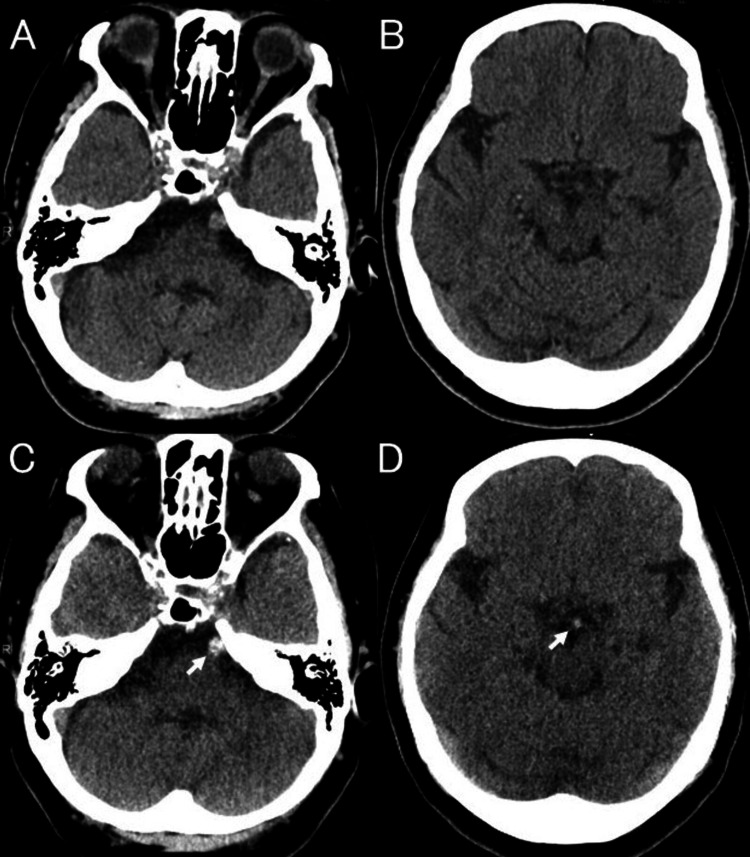
(A,B) Previous CT images compared with (C,D) noncontrast-enhanced CT obtained three hours after symptom onset reveal an HDBA sign (arrows) CT: computed tomography; HDBA: hyperdense basilar artery

This scan revealed the HDBA sign compared with the previously obtained CT images from the cerebral infarction two months ago (Figure 2).

**Figure 3:**
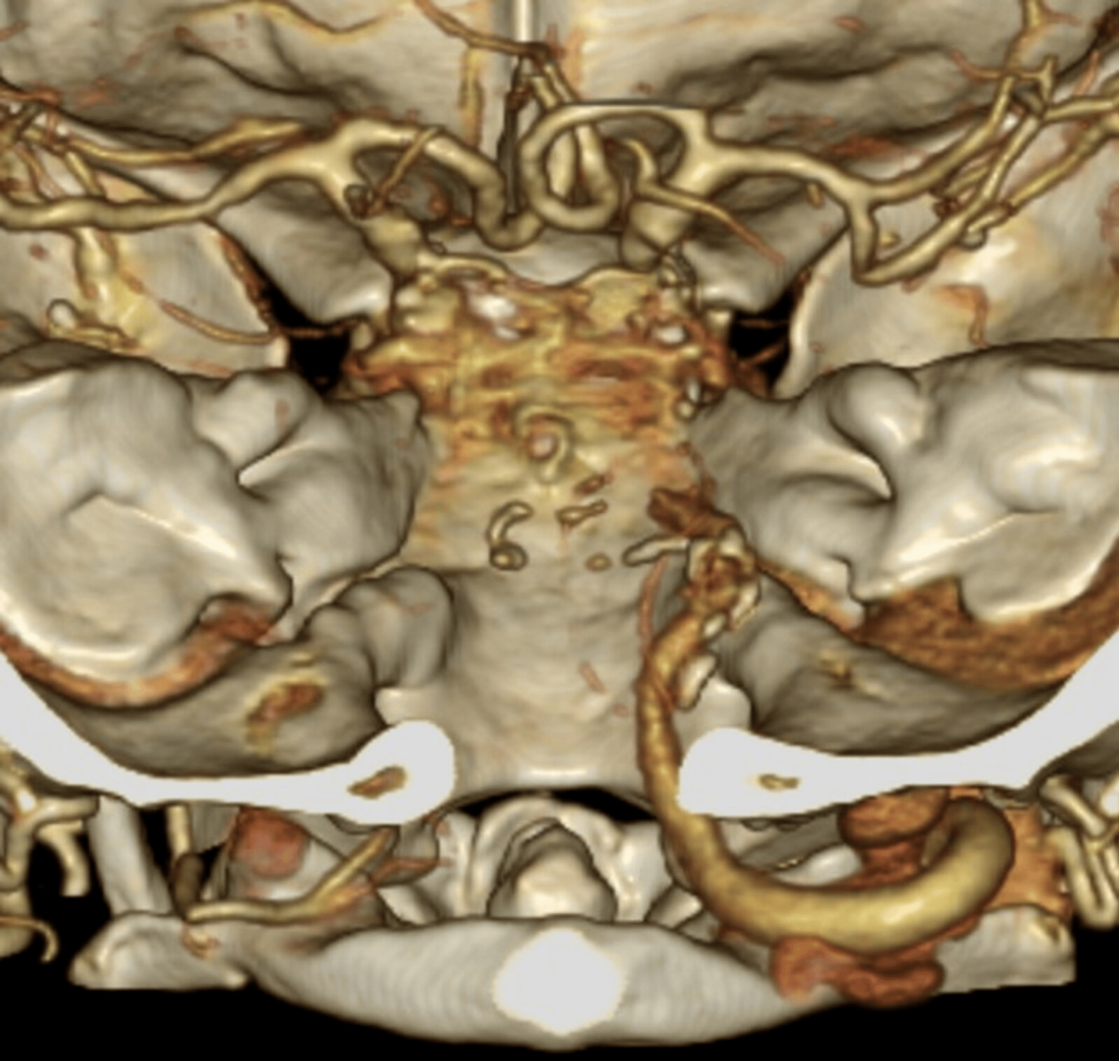
Three-dimensional CT angiography revealing a complete occlusion of the vertebrobasilar artery CT: computed tomography

Three-dimensional CT angiography revealed a complete vertebrobasilar artery occlusion (Figure 3).

No atrial fibrillation was detected on electrocardiography. Thrombectomy was performed to treat ischemic cerebrovascular disease due to BAO. Partial recanalization was achieved; however, significant basilar artery stenosis was noted. Antiplatelet therapy and neuroprotective treatment were administered intravenously. Spontaneous breathing resumed after treatment; however, the patient remained in a coma. The patient had a severe stroke and a modified Rankin Scale score of 6 on day 32.

## Discussion

The HDBA sign has been observed in 35.4%-71% of patients with posterior circulation ischemic stroke, indicating both BAO and brainstem infarction [2-5]. It is associated with more severe initial neurological deficits, larger infarct areas, and poorer functional outcomes [2-5]. The HDBA sign is a valuable tool for diagnosing BAO, with a sensitivity range of 54%-94% and a specificity range of 55%-98% [1,2,5,6]. However, significant interobserver variability exists in qualitative assessments of the HDBA sign [1].

Quantitative Hounsfield unit (HU) measurement is consistent across all digital imaging and medical reading software. It significantly enhances the specificity for identifying BAO with a consistent cutoff value, although sensitivity remains low [1,7]. The cutoff value for identifying HDBA varies from 40 to 61 HU in different studies [1,5,6].

In clinical practice, the HDBA sign is less well-established than the hyperdense MCA sign. This was because of concerns that posterior fossa artifacts may alter vessel density and the absence of comparable (paired) arteries for symmetry assessment. In this case, the basilar artery showed a hyperdense change compared with previous head CT images, and quantitative HU measurements showed elevated HU values. The HDBA sign is easier to evaluate qualitatively and quantitatively than previous head CT images.

Acute BAO is one of the most severe types of stroke and sometimes presents insidiously with unstable signs of brainstem dysfunction, such as dizziness, vomiting, dysphagia, dysarthria, ataxia, alternating hemiplegia, and gait instability, or acutely with coma and cardiac arrest [8-10]. Symptoms of BAO are often nonspecific to stroke compared to internal carotid artery occlusion or MCA occlusion, which can lead to a delay in diagnosis. In smaller hospitals and developing countries, equipment for magnetic resonance imaging (MRI) and the feasibility of MRI evaluation for posterior circulation ischemic stroke are limited because of artifacts and constraints associated with uncooperative or critically ill patients. Early detection of the HDBA sign on noncontrast-enhanced head CT followed by CT angiography, magnetic resonance angiography, or digital subtraction angiography is critical for expediting the diagnosis of BAO and maximizing the potential for neurological recovery.

## Conclusions

This case highlights the importance of comparing current and previous imaging to improve the detection of the HDBA sign. Quantitative HU measurement may further support the diagnosis. Early detection of the HDBA sign on noncontrast-enhanced head CT is critical for expediting the diagnosis and treatment of BAO. If a high specificity for detecting the HDBA sign is observed in comparison with previous CTs, a diagnosis of BAO can be made, allowing for the timely initiation of thrombolysis or thrombectomy. Future research should aim to elucidate the sensitivity and specificity of the HDBA sign through comparative analysis with prior CT imaging.
